# Supplementary material for: ERRγ enhances cardiac maturation with T-tubule formation in human iPSC-derived cardiomyocytes
Source: Nat Commun. 2021 Jun 21;12:3596. doi: 10.1038/s41467-021-23816-3 (PMC8217550; doi:10.1038/s41467-021-23816-3)
Supplement: Supplementary file 1 — Supplementary Information [file 41467_2021_23816_MOESM1_ESM.pdf]

Supplementary Figures

Supplementary Figure 1

Generation of double reporter hiPSC lines

Supplementary Figure 2

Compound screening information

Supplementary Figure 3

Expression profiles related to cardiac maturation in hiPSC-CMs.

Supplementary Figure 4

T112 accelerates metabolic and sarcomere maturation and contractile properties.

Supplementary Figure 5

Ultrastructural properties of hiPSC-CMs treated with DMSO, T112, T623 or Combo.

Supplementary Figure 6

Targeted homologous recombination strategy using the CRISPR/Cas9 system and analysis of the cardiac maturation status

Supplementary Table 1

Set of nucleases and gRNAs.

Supplementary Table 2

Primer sets for ddPCR and PCR.

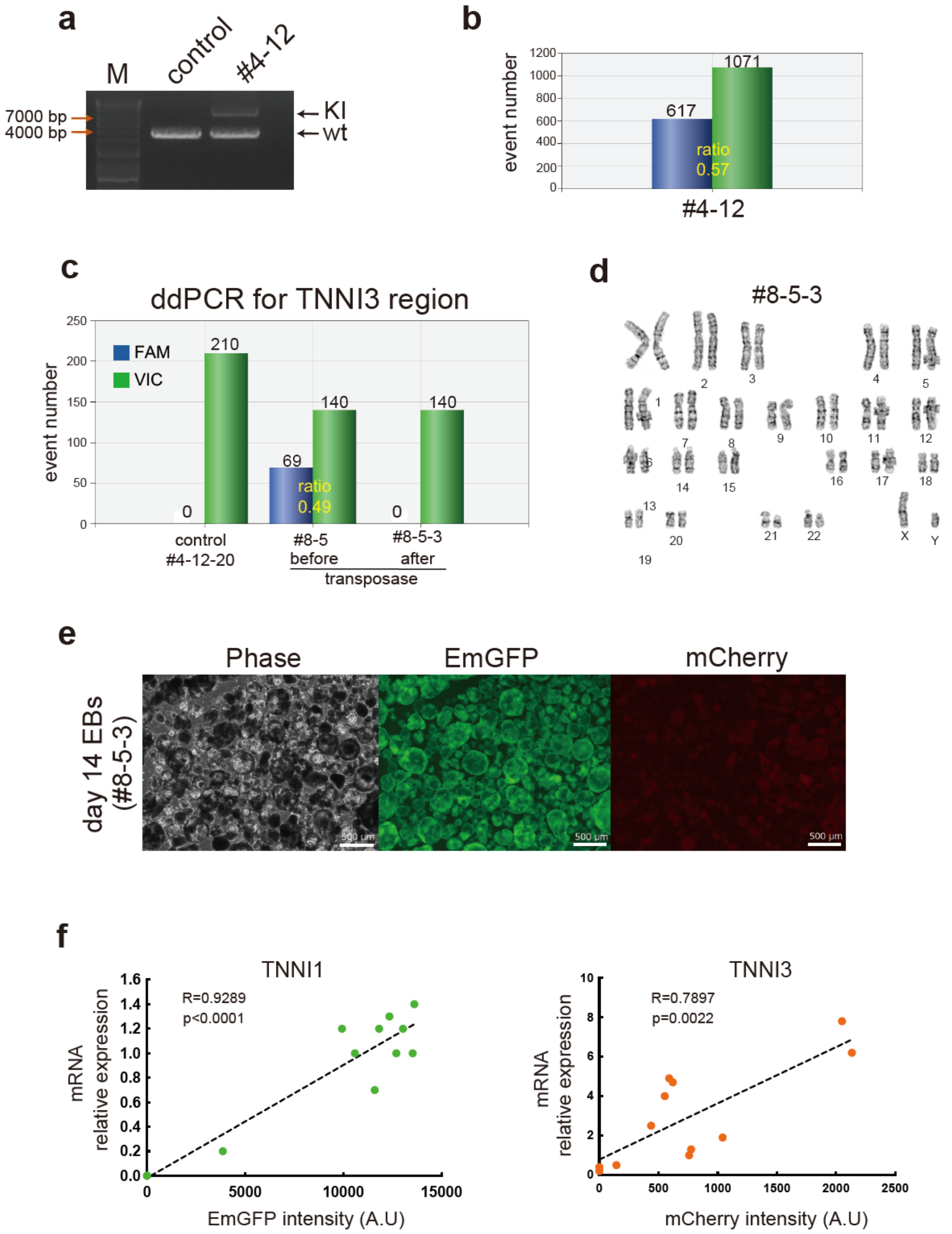

Supplementary Figure 1 Generation of double reporter hiPSC lines. **a**, PCR analysis of the TNNI1 targeted locus indicated in Figure 1A, black arrows. The primer sets used are listed in Supplementary Table 2. **b**, ddPCR of the TNNI1-EmGFP knockin clones before the removal of the transposons. **c**, ddPCR of the TNNI3-mCherry knockin clones before and after treatment with piggyBac

transposase. **d**, Karyotype of TNNI1-EmGFP knockin clone #8-5-3. **e**, Phase-contrast and EmGFP and mCherry fluorescence images of day-14 EBs. Scale bars: 500 µm. **f**, The relationship between TNNI1 expression and EmGFP intensity (**left**) and between TNNI3 expression and mCherry intensity (**right**). Pearson correlation was used to analyze the correlation between gene expressions and fluorescence intensities, and t-test (two-tailed) was used to test the null hypothesis: $r=0.9289$, $p<0.0001$ (left) and $r=0.7897$, $p=0.0022$ (right). Samples: day (d)0 hiPSCs, d3-no treatment (NT), d6-NT, d8-NT, d10-NT, d10-DMSO, d10-T112, d16-NT, d16-DMSO, d16-T112, d16-T623 and d16-Combo. Each dot indicates the mean of each sample. n=3 independent experiments per sample. The expression level of d16-DMSO = 1.0.

a

Plate Layout

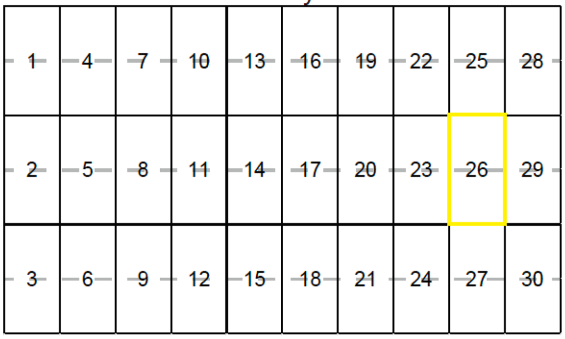

Treatment group (n=2)

1. w/o compound
2. vehicle (0.3% DMSO)
3. phenylephrine (PE, 10 μM)
4. Endothelin-1 (ET-1, 10 nM)
5. Isoproterenol (10 nM)
6. Metroprolol (100 nM)
7. Ivabradine (1 μM)
8. Isoproterenol (10 nM) + Ivabradine (1 μM)
9. KN-93 (1 μM)
10. Tryphostin 46 (1 μM)
11. Kenpaullone (1 μM)
12. Protein Kinase C inhibitor (PKCi, GF109203X, 1 μM)
13. Roscovitine (1 μM)
14. HDBA (1 μM)
15. PE (10 μM) + IL-6 100 ng/ml)
16. PE (10 μM) + KN-93 (1 μM)
17. PE (10 μM) + Tryphostin 46 (1 μM)
18. PE (10 μM) + Kenpaullone (1 μM)
19. PE (10 μM) + GF109203X (1 μM)
20. PE (10 μM) + Roscovitine (1 μM)
21. PE (10 μM) + HDBA (1 μM)
22. PE (10 μM) + Tryphostin 46 (1 μM) + Kenpaullone (1 μM)
23. ET-1 (10 nM) + KN-93 (1 μM)
24. ET-1 (10 nM) + Tryphostin 46 (1 μM)
25. ET-1 (10 nM) + Kenpaullone (1 μM)
26. ET-1 (10 nM) + GF109203X (1 μM)
27. ET-1 (10 nM) + Roscovitine (1 μM)
28. ET-1 (10 nM) + HDBA (1 μM)
29. ET-1 (10 nM) + Tryphostin 46 (1 μM) + Kenpaullone (1 μM)
30. VEGF (10 ng/ml)

b

EmGFP

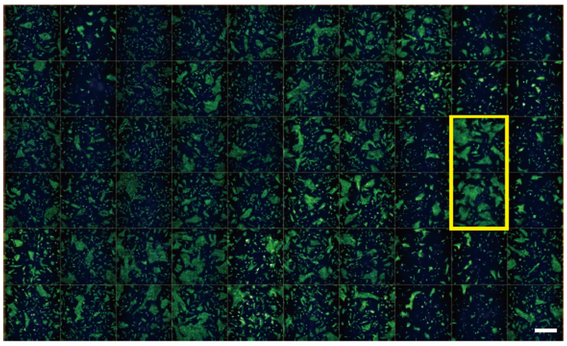

c

mCherry

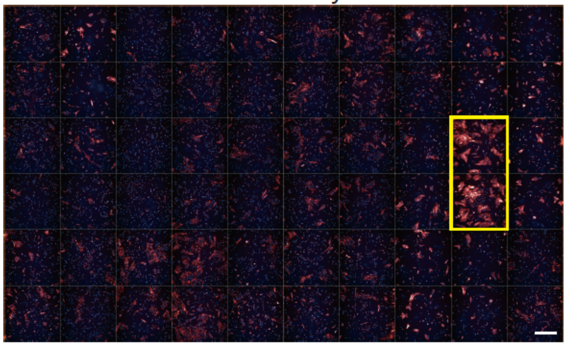

d

Type(s)	Unique Target
enzyme	457
kinase	431
G-protein coupled receptor	192
ion channel	141
other	131
peptidase	116
transporter	111
transcription regulator	81
transmembrane receptor	54
ligand-dependent nuclear receptor	37
phosphatase	30
cytokine	9
growth factor	9
translation regulator	5
Total	1804

e

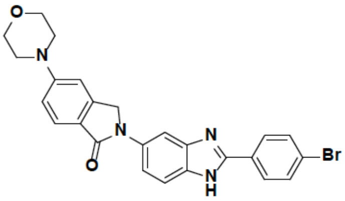

f

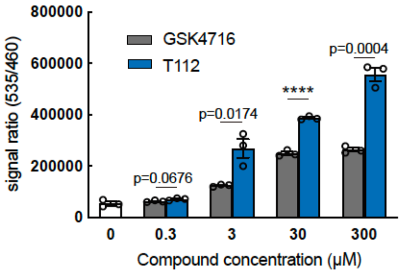

Supplementary Figure 2 Compound screening information.

a, **left**: Plate layout of the assay for determining the positive control. **right**: List of factors used in the assay. **b**, EmGFP images of the assay after treatment. Scale bars: 500 μm. **c**, mCherry images of the assay after treatment. Scale

bars: 500 μm. **d**, Our biologically annotated compound library. **e**, The chemical structural formula of T112, 2-[2-(4-bromophenyl)-1H-benzimidazol-5-yl]-5-(morpholin-4-yl)-2,3-dihydro-1H-isoindol-1-one. **f**, The TR-FRET PGC1a binding assay using T112 and GSK4716. n=3 independent experiments per group. Statistical analysis was done using unpaired two-tailed t-test. ****$P < 0.0001$.

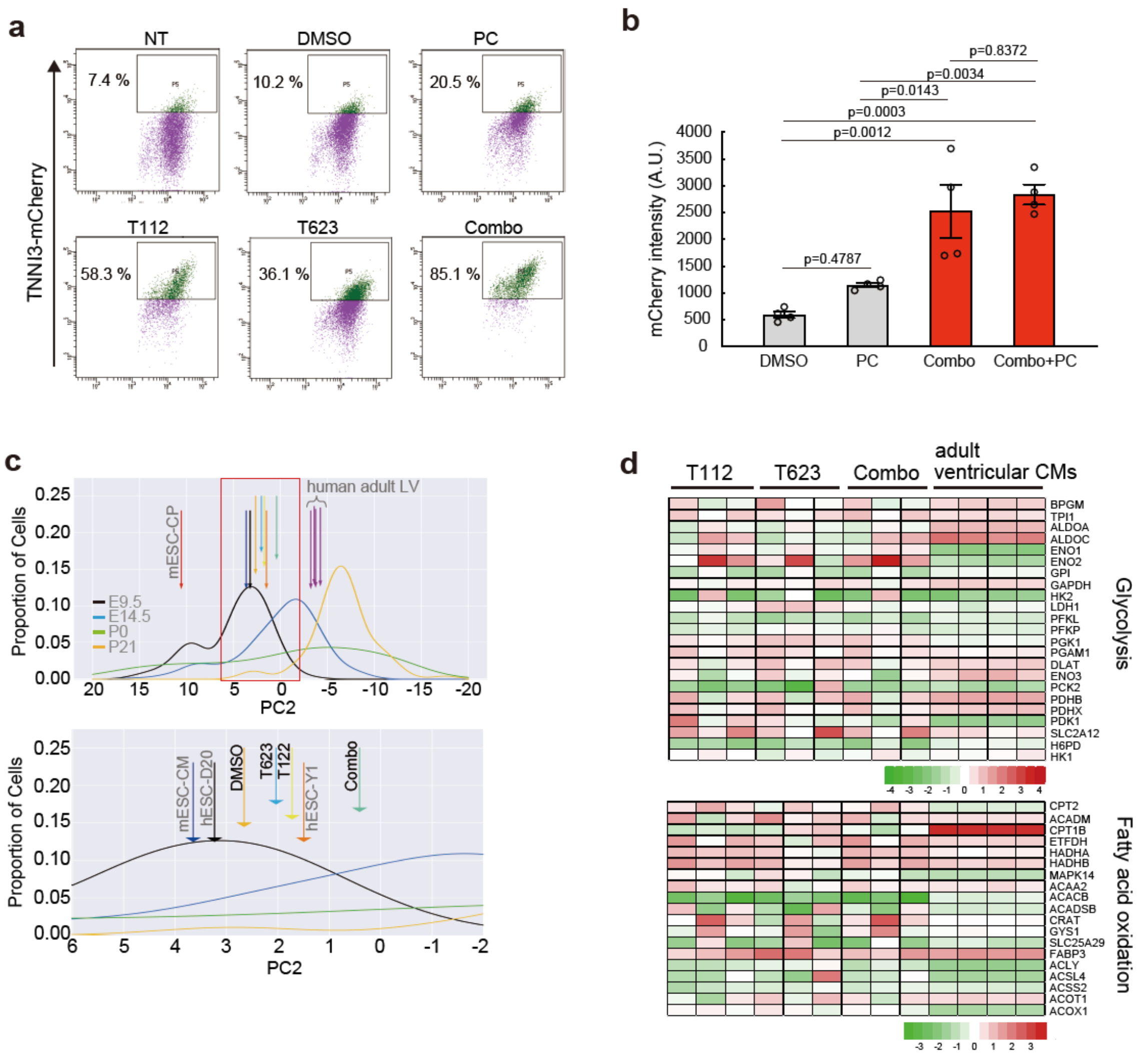

Supplementary Figure 3 Expression profiles related to cardiac maturation in hiPSC-CMs.

a, Representative flow cytometry images of the TNNI3-mCherry positive percentage of hiPSC-CMs treated with DMSO, positive control (PC), T112, T623 or both (Combo). **b**, Flow cytometric analysis of hiPSC-CMs treated with DMSO, PC, Combo or Combo+PC. n=4 independent experiments per group. Data are the mean ± SEM. Statistical analysis was done using one-way ANOVA followed by Tukey's HSD test. **c**, top: Density plot (histogram) of the proportion of murine ventricular CMs at E9.5, E14.5, p0 and p21 based on previously published PCA[1] and transcriptional analyses of the datasets obtained in this study using day-16 hiPSC-CMs treated with DMSO (orange arrow), T623 (aqua arrow), T112 (yellow arrow) or T623 and T112 (Combo, green arrow) and public datasets of mESC-CP (red arrow), mESC-CM (blue arrow), hESC-D20 (black

arrow), hESC-Y1 (brown arrow) and adult ventricular CMs (purple arrow)[2, 3]. bottom: Magnification of the red box in top. **d**, Heatmap of the expression changes in glycolysis- and fatty acid oxidation-related genes. Data are normalized to the DMSO-treated samples.

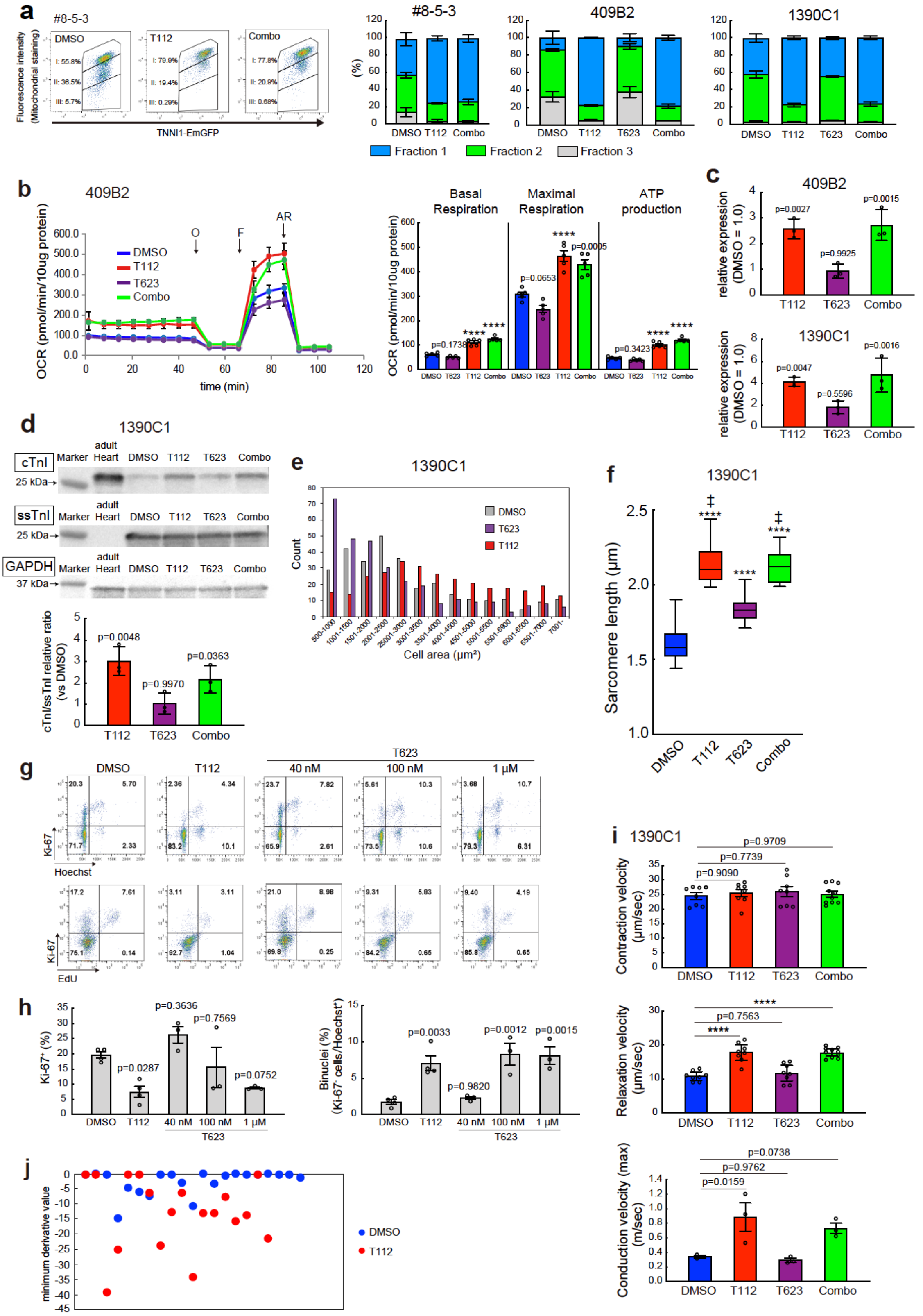

Supplementary Figure 4 T112 accelerates metabolic and sarcomere maturation and contractile properties.

a, **left**: Representative flow cytometry images of hiPSC (#8-5-3)-CMs treated with DMSO, T112 or Combo stained with mitochondrial dyes. **right**: Percentage of each fraction in each group of hiPSC-CMs in multiple hiPSC lines (#8-5-3, 409B2 and 1390C1). n=3 independent experiments per group. Data are the mean ± SEM. **b**, **left**: Mitochondrial respiration rates of hiPSC (409B2)-CMs treated with DMSO, T112, T623 or Combo. n=5 biologically independent samples per group. Data are the mean ± SEM. **right**: Basal and maximal OCR and ATP production. n=5 biologically independent samples per group. Statistical analysis was compared to DMSO using one-way ANOVA followed by Dunnett's test. ****$P < 0.0001$. **c**, TNNI3 expression level in hiPSC-CMs treated with DMSO, T112, T623 or Combo. **top**: 409B2, **bottom**: 1390C1. n=3 independent experiments per group. Data are the mean ± SEM. Statistical analysis was compared to DMSO using one-way ANOVA followed by Dunnett's test. **d**, **top**: Representative western blots of cTnI, ssTnI and GAPDH proteins in hiPSC (1390C1)-CMs treated with DMSO, T112, T623 or Combo. **bottom**: cTnI/ssTnI relative ratio. n=3 independent experiments per group. Data are the mean ± SEM. Statistical analysis was compared to DMSO using one-way ANOVA followed by Dunnett's test. **e**, Cell area of hiPSC (1390C1)-CMs treated with DMSO, T112 or T623 measured by high content imaging. For each group, n=300 cells over 3 independent experiments. **f**, Sarcomere length in hiPSC (1390C1)-CMs treated with DMSO, T112, T623 or Combo. Boxes represent 25th–75th percentiles; whiskers represent the minimum and maximum ranges; horizontal lines indicate the median values. n=15, one measurement per TEM image from 15 TEM images over 2 consecutive experiments; ****$P < 0.0001$ compared to DMSO, ‡$P < 0.0001$ compared to T623 using one-way ANOVA followed by Tukey's HSD test. **g**, Representative flow cytometry images of day-16 cells stained with Ki-67, EdU and Hoechst. **h**, Percentage of Ki-67^{+} (**left**) and Ki-67^{-}/Hoechst^{+} cells (**right**) in hiPSC (#8-5-3)-CMs treated with DMSO (n=4), T112 (n=4) or T623 (n=3). n=independent experiments per group. Data are the mean ± SEM. Statistical analysis was compared to DMSO using one-way ANOVA followed by Dunnett's test. **i**, Contractile properties of hiPSC (1390C1)-CMs treated with DMSO (n=8), T112 (n=8), T623 (n=8) or Combo (n=10). **top**: Contraction velocity. **middle**: Relaxation velocity. n=biologically independent samples examined over 3 independent experiments. **bottom**: Maximum conduction velocity. n=3 independent experiments. Data are the mean ± SEM. Statistical analysis was compared to DMSO using one-way ANOVA followed by

Dunnett's test. ****P < 0.001. **j**, The minimum derivative value of AP traces in hiPSC (#8-5-3)-CM treated with DMSO (blue, n=21) or T112 (red, n=18) over 3 independent experiments.

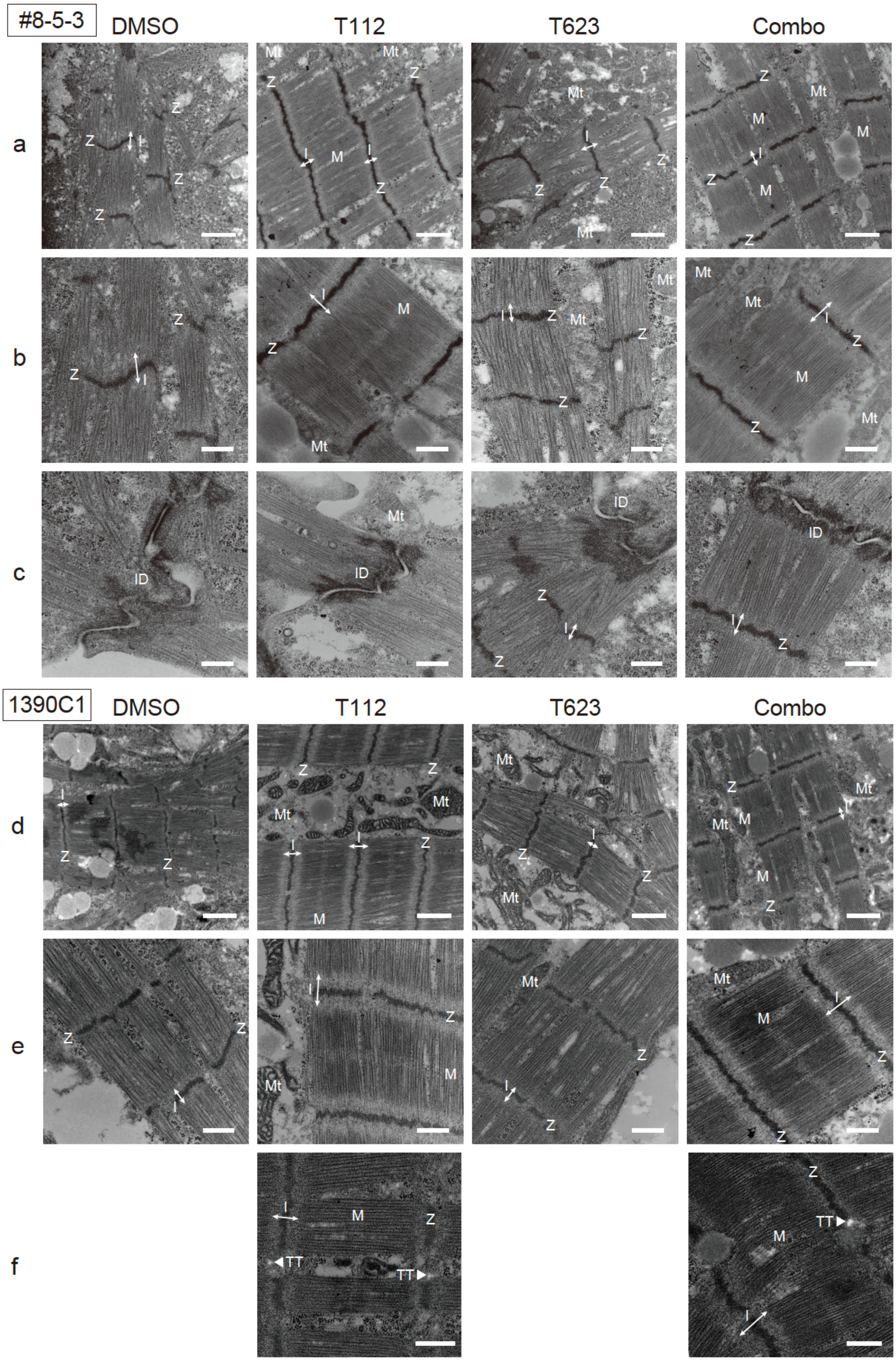
#8-5-3
DMSO
T112
T623
Combo
a
b
c
1390C1
DMSO
T112
T623
Combo
d
e
f
Z
I
M
Mt
ID
TT

Supplementary Figure 5 Ultrastructural properties of hiPSC-CMs treated with DMSO, T112, T623 or Combo.

a-c, #8-5-3. **d**-**f**, 1390C1.

a and **d**, Typical low magnification TEM images. Scale bars: 1000 nm. **b**, **c**, **e** and **f**, Typical high magnification TEM images. Scale bars: 500 nm. I: I-band; ID: intercalated disc; M: M-line; Mt: Mitochondria; TT: T-tubule; Z: Z line.

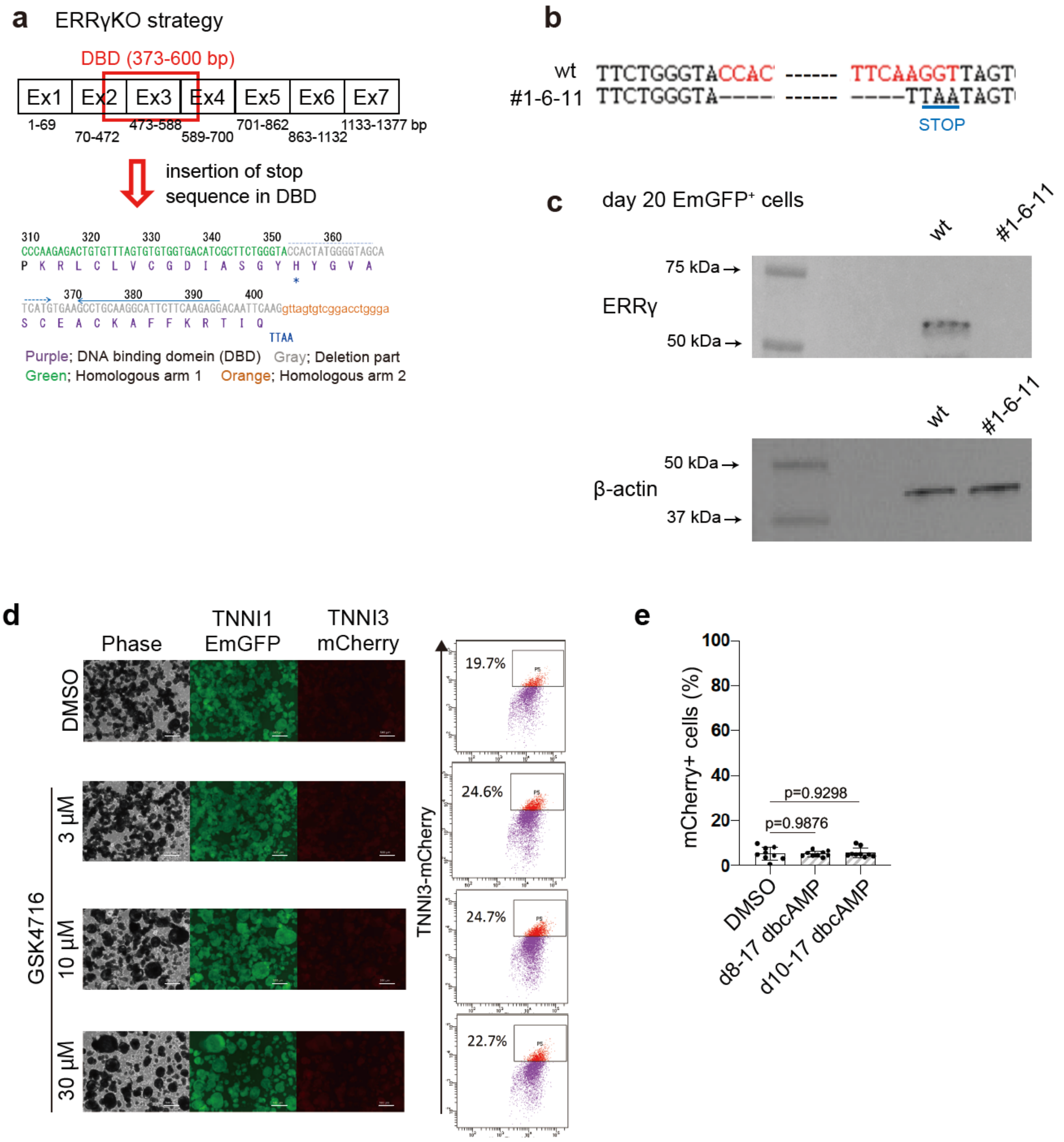

Supplementary Figure 6 Targeted homologous recombination strategy using the CRISPR/Cas9 system and analysis of the cardiac maturation status. **a**, The ERRγKO strategy. **b**, Sequence analysis of the targeted region. **c**, Western blotting of ERRγ and β-actin in day-20 CMs derived from WT or KO (#1-6-11) clones. **d**, **left**: Fluorescence images of day-16 EBs treated with GSK4716. Scale bars: 500 μm. **right**: Representative flow cytometry images of hiPSC-CMs treated with DMSO or GSK4716. TNNI3-mCherry positive percentages are shown. **e**, Flow cytometric analysis of ERRγ KO hiPSC-CMs treated with 0.5 mM dbcAMP from day 8 to 17 or from day 10 to 17. n=9 biologically independent samples examined over 3 independent experiments.

Data are the mean ± SEM. Statistical analysis was done using one-way ANOVA followed by Dunnett’s test.

Supplementary Table 1. Set of nucleases and gRNAs.

	Nuclease	gRNA	
		name	sequence
TNNI1-EmGFP	Cas9 (D10A)	AS194	CTCTAGGTACCTCTATTGTGAGG
		S254	TGGGGTCCATCAGAGTCTAGAGG
TNNI3-mCherry	Cas9 VQR variant	S4	CAAGAAAAAGTTTGAGAGCTGA
ERRγ KO	Cas9 (D10A)	AS345	CCACTATGGGGTAGCATCATG
		S393	GCCTGCAAGGCATTCTTCAAGAGG

Supplementary Table 2 Primer sets for ddPCR and PCR.

ddPCR TNNI1	TNNI1out_F1021	GGGCGCTTCTCACCTACAGAT
	TNNIout_P1043 (VIC)	AAGGACCTGAAGCTGAA
	TNN1out_R1083	AACTTCCCACGGAGGTCCA
ddPCR TNNI3	TNNI3_HA2out_Fwd	AGCTAGTCAGCATCTGGCAATG
	TNNI3_HA2out_probe (VIC)	ATGGCTGCAATGGTT
	TNNI3_HA2out_Rev	CCGGCCTCAAGATAAGCAATAT
ddPCR donor vector	Donor_upTaqF345-363	GGCACTTGGCGCTACACAA
	Donor_TaqP368-382 (FAM)	CCTCTGGCCTCGCAC
	Donor_upTaqR404-385	CCTACCGGTGGATGTGGAAT
PCR TNNI1	TNNI1outF1	GGCTTCAAGGGTGGACAATTTAAGGTG
	TNNI1outR4	TGCAGCGCATGAATCCCTGACTTAC
PCR TNNI3	TNNI3 dPCR Fwd	GATGCCTGAAACCATGGATTG
	TNNI3 HA2 out R	CCGGCCTCAAGATAAGC
PCR ERRγKO	ESRRGoutF3	GAAACAAAGTGATTGAGGATATATTCCC
	HA2-nest-Rv	CCTCATCTATAAATTGAAGGCATT

References

1. DeLaughter, D.M. *et al.* Single-Cell Resolution of Temporal Gene Expression during Heart Development. *Dev Cell* **39**, 480-490 (2016).
2. Wamstad, J.A. *et al.* Dynamic and coordinated epigenetic regulation of developmental transitions in the cardiac lineage. *Cell* **151**, 206-220 (2012).
3. Kuppusamy, K.T. *et al.* Let-7 family of microRNA is required for maturation and adult-like metabolism in stem cell-derived cardiomyocytes. *Proc Natl Acad Sci U S A* **112**, E2785-2794 (2015).
