# Supplementary material for: ERRγ enhances cardiac maturation with T-tubule formation in human iPSC-derived cardiomyocytes
Source: Nat Commun. 2021 Jun 21;12:3596. doi: 10.1038/s41467-021-23816-3 (PMC8217550; doi:10.1038/s41467-021-23816-3)
Supplement: Supplementary file 3 — Reporting Summary [file 41467_2021_23816_MOESM3_ESM.pdf]

nature research

Corresponding author(s): Yoshinori Yoshida and Tomoyuki Nishimoto

Last updated by author(s): Apr 22, 2021

Reporting Summary

Nature Research wishes to improve the reproducibility of the work that we publish. This form provides structure for consistency and transparency in reporting. For further information on Nature Research policies, see Authors & Referees and the Editorial Policy Checklist .

Statistics

For all statistical analyses, confirm that the following items are present in the figure legend, table legend, main text, or Methods section.

n/a	Confirmed	
☐	☒	The exact sample size (n) for each experimental group/condition, given as a discrete number and unit of measurement
☐	☒	A statement on whether measurements were taken from distinct samples or whether the same sample was measured repeatedly
☐	☒	The statistical test(s) used AND whether they are one- or two-sided<br>*Only common tests should be described solely by name; describe more complex techniques in the Methods section.*
☒	☐	A description of all covariates tested
☐	☒	A description of any assumptions or corrections, such as tests of normality and adjustment for multiple comparisons
☐	☒	A full description of the statistical parameters including central tendency (e.g. means) or other basic estimates (e.g. regression coefficient) AND variation (e.g. standard deviation) or associated estimates of uncertainty (e.g. confidence intervals)
☐	☒	For null hypothesis testing, the test statistic (e.g. F, t, r) with confidence intervals, effect sizes, degrees of freedom and P value noted<br>*Give P values as exact values whenever suitable.*
☒	☐	For Bayesian analysis, information on the choice of priors and Markov chain Monte Carlo settings
☒	☐	For hierarchical and complex designs, identification of the appropriate level for tests and full reporting of outcomes
☒	☐	Estimates of effect sizes (e.g. Cohen's d, Pearson's r), indicating how they were calculated

Our web collection on statistics for biologists contains articles on many of the points above.

Software and code

Policy information about availability of computer code

Data collection	RNA-seq: llumina Nextseq 500<br>High-content images: OperaPhenix imager (Perkin Elmer)<br>Fluoresence Microscopy: BZ-X700 (Keyence)<br>Confocal Microscopy: LSM880 (Zeiss)<br>Flow Cytometry: BD FACSDiva (BD)<br>Wester Blot: Lumino Graph (ATTO)<br>qPCR: QuantStudio 7 Flex (ThermoFisher)<br>Mitochondria function assay: Seahorse Bioscience XF24 extracellular flux analyzer (agilent)<br>Motion vector analysis: SI8000 (Sony) and SI8000 View Software (v1.05.0108)<br>Patch clamp: Axopatch 200B amplifier (Molecular Devices), DigiData (1322A) and pCLAMP(v9.2)<br>Transmission electron microscopy: Hitachi H-7650 (Hitachi)
Data analysis	RNA-seq: R (3.6.1), NOISeq (2.28.0), STAR (2.5.4a), Python (3.7.0)<br>Flow cytometric analysis: BD FACSDiva (v.8.0.1) and FlowJo (v10.5.3)<br>Imaging: ImageJ (v 1.51m9)<br>Mitochondria function assay: wave software (v2.6.1)<br>Motion vector analysis:SI8000C Analyzer software (v1.05.0105)<br>Patch clamp: pCLAMP(v10.7)<br>Roundness: Harmony software (v4.9)<br>Statistical analysis: GraphPad Prism (v8.4.3)<br>TR-FRET: Envision Manager (v.1.13)

For manuscripts utilizing custom algorithms or software that are central to the research but not yet described in published literature, software must be made available to editors/reviewers. We strongly encourage code deposition in a community repository (e.g. GitHub). See the Nature Research guidelines for submitting code & software for further information.

Data

Policy information about availability of data

All manuscripts must include a data availability statement. This statement should provide the following information, where applicable:

- Accession codes, unique identifiers, or web links for publicly available datasets
- A list of figures that have associated raw data
- A description of any restrictions on data availability

The RNA-seq data reported in this paper have been deposited in NCBI's Gene Expression Omnibus and are accessible through GEO series accession number GSE135319. The data that support the findings of this study are available from the corresponding author upon reasonable request.
The mouse single cell RNA-seq data are publicly available from the GNomEx database (accession numbers: 272R, 274R, 275-292R, 439R, and 440R). mESC-CP, mESC-CMs, hESC-D20, h-ESCY1 and heart tissue data are publicly available from the NCBI GEO databank (accession numbers: GSE47948, GSE62913 and GSE46224).

Field-specific reporting

Please select the one below that is the best fit for your research. If you are not sure, read the appropriate sections before making your selection.

☒ Life sciences ☐ Behavioural & social sciences ☐ Ecological, evolutionary & environmental sciences

For a reference copy of the document with all sections, see nature.com/documents/nr-reporting-summary-flat.pdf

Life sciences study design

All studies must disclose on these points even when the disclosure is negative.

Sample size	Required experimental sample sizes were estimated based on previous established protocols in the field (e.g. Kattman S. J. et al., Cell Stem Cell 2011, Ronaldson-Bouchard K. et al., Nature 2018). The sample sizes were adequate as the differences between experimental groups were reproducible. All n values are clearly indicated within the figure legends.
Data exclusions	No data were excluded for the analyses
Replication	All experiments were repeated multiple times as indicated in each figure legend.
Randomization	Randomization is not applicable to this study because we did not perform any experiments where there are treatment and control groups that would necessitate randomization between the subjects.
Blinding	Blinding is not applicable to this study because we did not perform any experiments where there are treatment and control groups that would necessitate blinding.

Reporting for specific materials, systems and methods

We require information from authors about some types of materials, experimental systems and methods used in many studies. Here, indicate whether each material, system or method listed is relevant to your study. If you are not sure if a list item applies to your research, read the appropriate section before selecting a response.

Materials & experimental systems

n/a	Involved in the study	
☐	☒	Antibodies
☐	☒	Eukaryotic cell lines
☒	☐	Palaeontology
☒	☐	Animals and other organisms
☒	☐	Human research participants
☒	☐	Clinical data

Methods

n/a	Involved in the study	
☒	☐	ChIP-seq
☐	☒	Flow cytometry
☒	☐	MRI-based neuroimaging

Antibodies

Antibodies used

Cardiac Troponin T Monoclonal Antibody (Thermo Fisher Scientific, MA5-12960, Clone 13-11, dilution 1:500)
mCherry Monoclonal Antibody (Thermo Fisher Scientific, M11217, Clone 16D7, dilution 1:200)
ERRγ antibody (Abcam, ab49129, dilution 1:2000)
Actin Monoclonal Antibody (Merck Millipore, MAB1501, Clone C4, dilution 1:5000)
GAPDH antibody (Cell Signaling, 5174, dilution 1:1000)
cTNT Monoclonal Antibody (Thermo, MS-295-P, Clone 13-11, dilution 1:500)
cardiac TroponinI (cTnI) Monoclonal Antibody (Thermo, MA1-20112, Clone 16A11, dilution 1:250)

	TroponinI1 (ssTnI) Antibody (abcam, EPR17120-11, dilution 1:1000)<br>Ki-67 Monoclonal Antibody (BD, 556003, Clone B56, dilution 1:200)<br>PE/Cyanine7 anti-human CD172a/b (SIRPα/β) Antibody (BioLegend, 323808, Clone SE5A5, 1:500)<br>APC Mouse Anti-Human CD90 (BD, 559869, Clone 5E10, 1:1000)<br>APC anti-human CD31 Antibody (BioLegend, 303116, Clone WM59, 1:500)<br>Alexa Fluor® 647 anti-human CD49a Antibody (BioLegend, 328310, Clone TS2/7, 1:500)<br>APC anti-mouse CD140b Antibody (BioLegend, 136008, Clone APB5, 1:500)<br>Goat anti-Rabbit IgG (H+L) Highly Cross-Adsorbed Secondary Antibody, Alexa Fluor 546 (Thermo Fisher Scientific, A-11035, dilution 1:500)<br>Goat anti-Rat IgG (H+L) Cross-Adsorbed Secondary Antibody, Alexa Fluor 647 (Thermo Fisher Scientific, A-21247, dilution 1:200)<br>Anti-mouse IgG, HRP-Linked Whole Ab Sheep (GE Healthcare, NA931, dilution 1:5000)<br>Anti-Rabbit IgG, HRP-Linked Whole Ab Donkey (GE Healthcare, NA934, dilution 1:5000)<br>APC Goat anti-mouse IgG (minimal x-reactivity) (biolegend, 405308, dilution 1:500)
Validation	Cardiac Troponin T (MA5-12960): Miki K, et al., Cell Stem Cell. 2015<br>mCherry: Shrestha RL, et al., Nature communications, 2017; Schifferer M, et al., Cell chemical biology. 2017<br>ERRγ: validated by the manufacture.<br>Actin: Frank CL, et al., Nature neuroscience, 2015; Yan Y, et al., Nature communications 2015<br>GAPDH: Yuxuan Wu, et al., Nature Medicine 2019; Tingting Li, et al., J Clin Invest. 2019<br>cTNT (MS-295-P): Malouf NN, et al., Journal of Biological Chemistry. 1992<br>cardiac TroponinI (cTnI): Nagy N, et al., Development 2016<br>TroponinI1 (ssTnI) : validated by the manufacture.<br>Ki-67: Starborg M., et al., J Cell Sci. 1996; Bigley V., et al., J Exp Med. 2011

Eukaryotic cell lines

Policy information about cell lines

Cell line source(s)	The 1390D4, 1390C1, and 409B2 hiPSC lines used in this study were generated in CiRA
Authentication	All hiPSC used in this study including reporter lines were validated with karyotype testing.
Mycoplasma contamination	All cell lines are regularly tested and were always negative for mycoplasma
Commonly misidentified lines<br>(See ICLAC register)	No commonly misidentified lines were used in this study.

Flow Cytometry

Plots

Confirm that:

- [x] The axis labels state the marker and fluorochrome used (e.g. CD4-FITC).
- [x] The axis scales are clearly visible. Include numbers along axes only for bottom left plot of group (a 'group' is an analysis of identical markers).
- [x] All plots are contour plots with outliers or pseudocolor plots.
- [x] A numerical value for number of cells or percentage (with statistics) is provided.

Methodology

Sample preparation	EBs were dissociated into single cells by Liberase and TrypLE Select. For sorting of CMs derived from #-8-5-3 hiPSC lines, dissociated cells were suspended in 2% FBS/PBS. For sorting of CMs derived from 1390C1 and 409B2 hiPSC lines, dissociated cells were stained with PE/Cyanine7 anti-human CD172a/b (SIRPα/β) Antibody (BioLegend, 323808, Clone SE5A5, 1:500), APC Mouse Anti-Human CD90 (BD, 559869, Clone 5E10, 1:1000), APC anti-human CD31 Antibody (BioLegend, 303116, Clone WM59, 1:500), Alexa Fluor® 647 anti-human CD49a Antibody (BioLegend, 328310, Clone TS2/7, 1:500) and APC anti-mouse CD140b Antibody (BioLegend, 136008, Clone APB5, 1:500). For Ki-67 staining, dissociated cells were stained with mouse anti-Ki-67 Clone B56 (RUO) (BD, 556003, 1:200 dilution) and APC Goat anti-mouse IgG (minimal x-reactivity) (biolegend, 405308, 1:500 dilution). For EdU assay, dissociated cells were using the Click-iTTM EdU Alexa FluorTM 647 Flow Cytometry Assay Kit.
Instrument	FACS Aria Fusion
Software	Acquisition: BD FACSDiva (v.8.0.1)<br>Analysis : Flowjo (v10.5.3)
Cell population abundance	Flow cytometry analysis populations were generally >5%.
Gating strategy	Cells were gated on FSC/SSC first and an unstained sample as controls to establish the gate for positive cells. Regarding TNNI3-mcherry gate, cells were gated on a DMSO-treated sample as control to establish the gate for positive cells. For sorting of CMs

derived from 1390C1 and 409B2 hiPSC lines, cells were gated on an unstained sample as control to establish the gate for positive cells. For Ki-67 staining and EdU assay, cells were gated on an unstained sample as control to establish the gate for positive cells.

☒ Tick this box to confirm that a figure exemplifying the gating strategy is provided in the Supplementary Information.
