# Supplementary material for: ERRγ enhances cardiac maturation with T-tubule formation in human iPSC-derived cardiomyocytes
Source: Nat Commun. 2021 Jun 21;12:3596. doi: 10.1038/s41467-021-23816-3 (PMC8217550; doi:10.1038/s41467-021-23816-3)
Supplement: Supplementary file 4 — Source Data [file 41467_2021_23816_MOESM4_ESM.zip › Source data WB v1.pdf]

Figure 5C

WB full scan images

cTnI

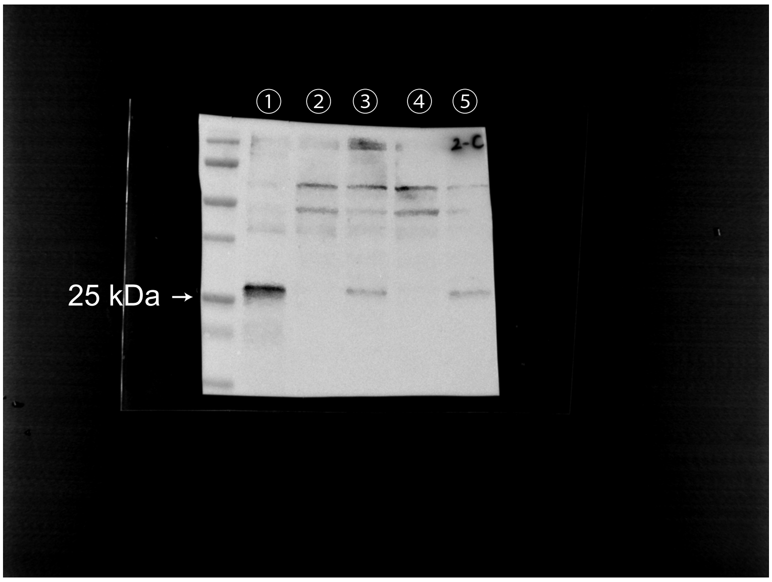

ssTnI

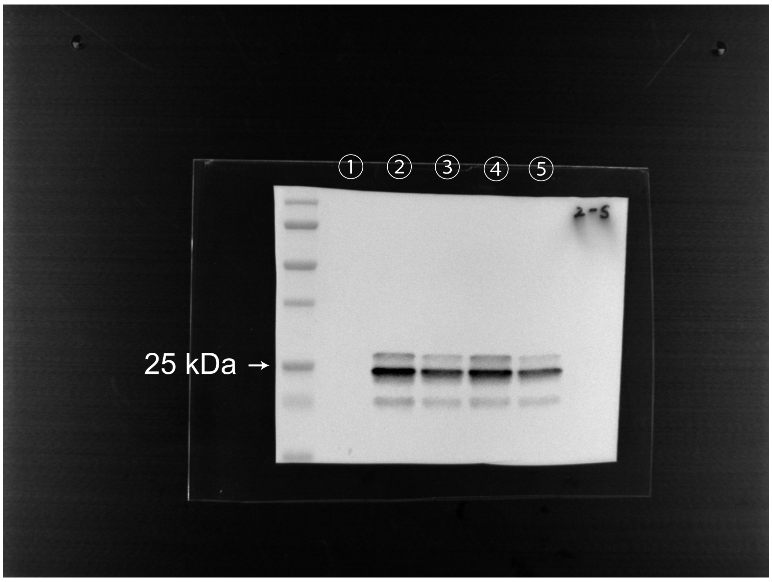

GAPDH

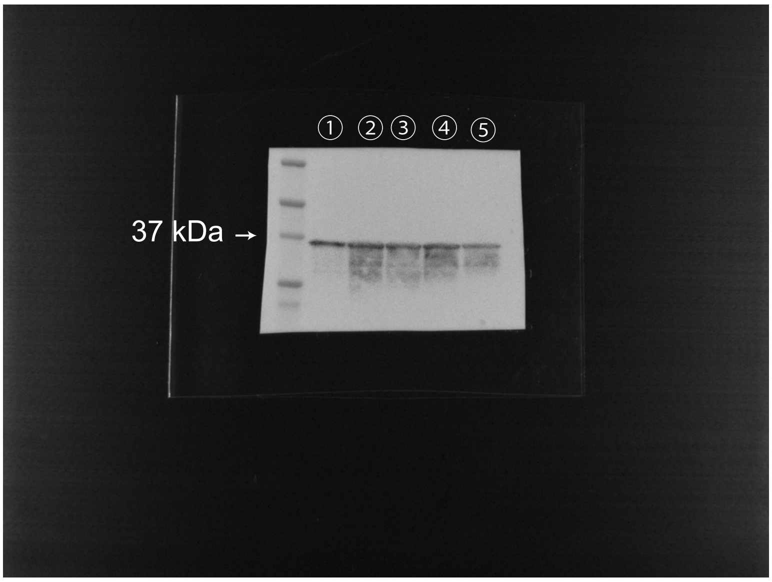

① adult Heart
② DMSO
③ T112
④ T623
⑤ Combo

Supplementary Figure 4d

WB full scan images

cTnI

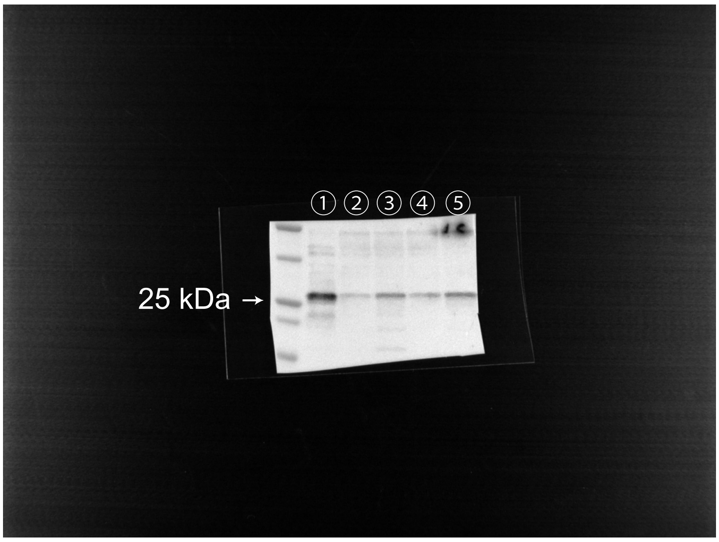

ssTnI

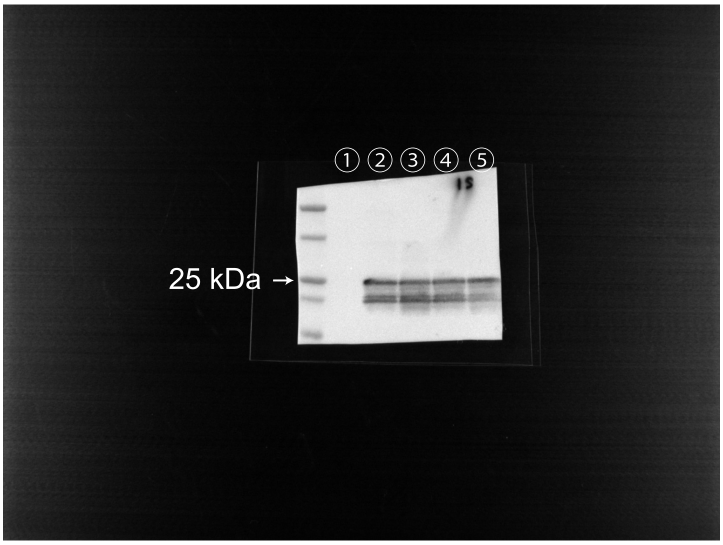

GAPDH

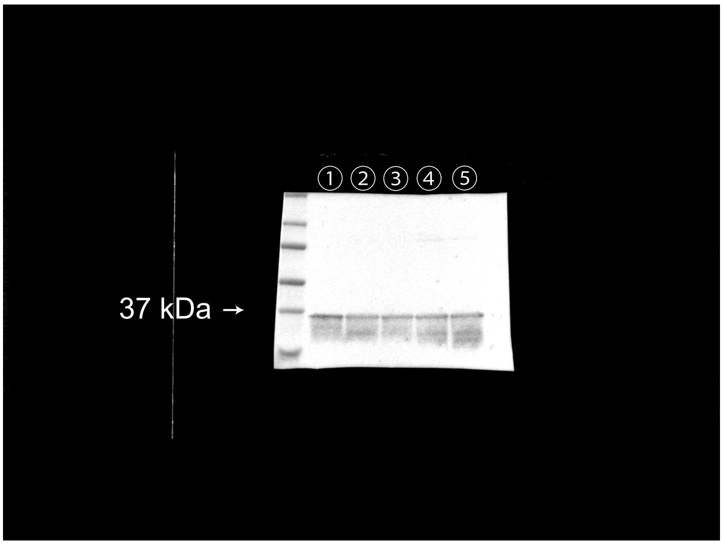

① adult Heart
② DMSO
③ T112
④ T623
⑤ Combo

Supplementary Figure 6c

WB full scan images

ERRγ

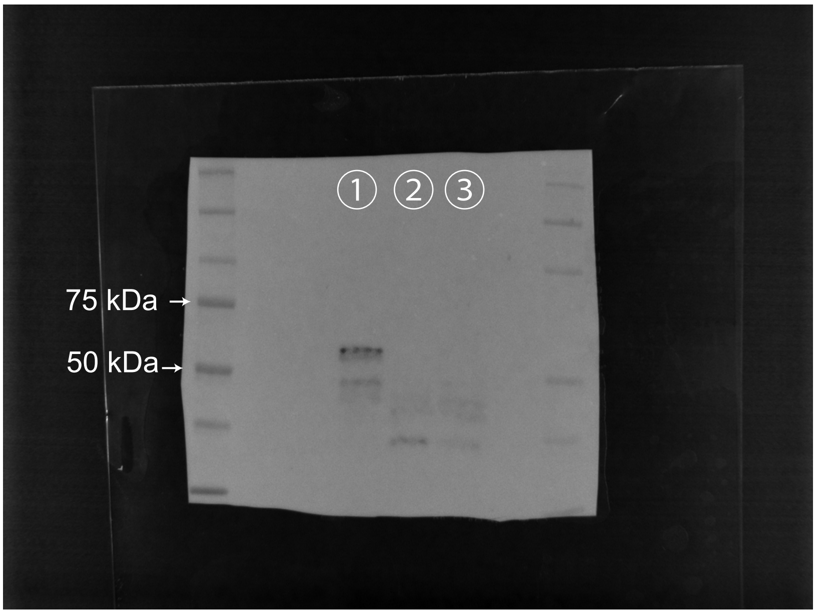

β-actin

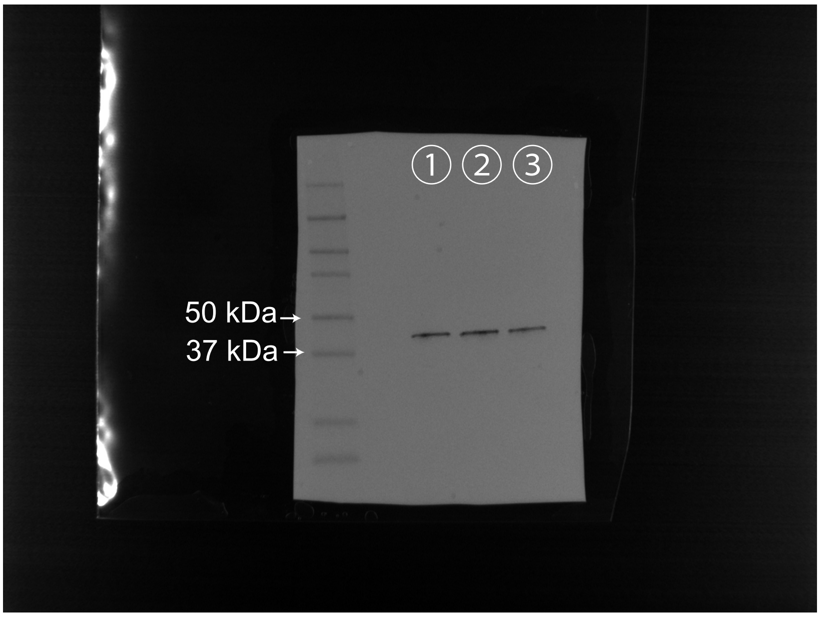

① WT

② #1-6-11 (KO line)

③ #1-11-4 (another KO line, we did not use it in this study)
